# C1 Complex: An Adaptable Proteolytic Module for Complement and Non-Complement Functions

**DOI:** 10.3389/fimmu.2017.00592

**Published:** 2017-05-24

**Authors:** Jinhua Lu, Uday Kishore

**Affiliations:** ^1^Department of Microbiology and Immunology, Yong Loo Lin School of Medicine and Immunology Programme, National University of Singapore, Singapore; ^2^Department of Life Sciences, College of Health and Life Sciences, Brunel University London, Uxbridge, UK

**Keywords:** complement C1, autoimmunity, aging, infection, inflammation, C1q, macrophage, dendritic cell

## Abstract

Complement C1 is the defining component of the classical pathway. Within the C1qC1r_2_C1s_2_ complex, C1q functions as a molecular scaffold for C1r_2_C1s_2_ and C1q binding to its ligands activates these two serine proteases. The classic C1q ligands are antigen-bound antibodies and activated C1s cleaves C4 and C2 to initiate the complement cascade. Recent studies suggest broad C1 functions beyond the complement system. C1q binds to the Frizzled receptors to activate C1s, which cleaves lipoprotein receptor-related protein 6 to trigger aging-associated Wnt receptor signaling. C1q binds to apoptotic cells and the activated C1 proteases cleave nuclear antigens. C1s also cleaves MHC class I molecule and potentially numerous other proteins. The diversity of C1q ligands and C1 protease substrates renders C1 complex versatile and modular so that it can adapt to multiple molecular and cellular processes besides the complement system.

## Introduction

**Figure 1:**
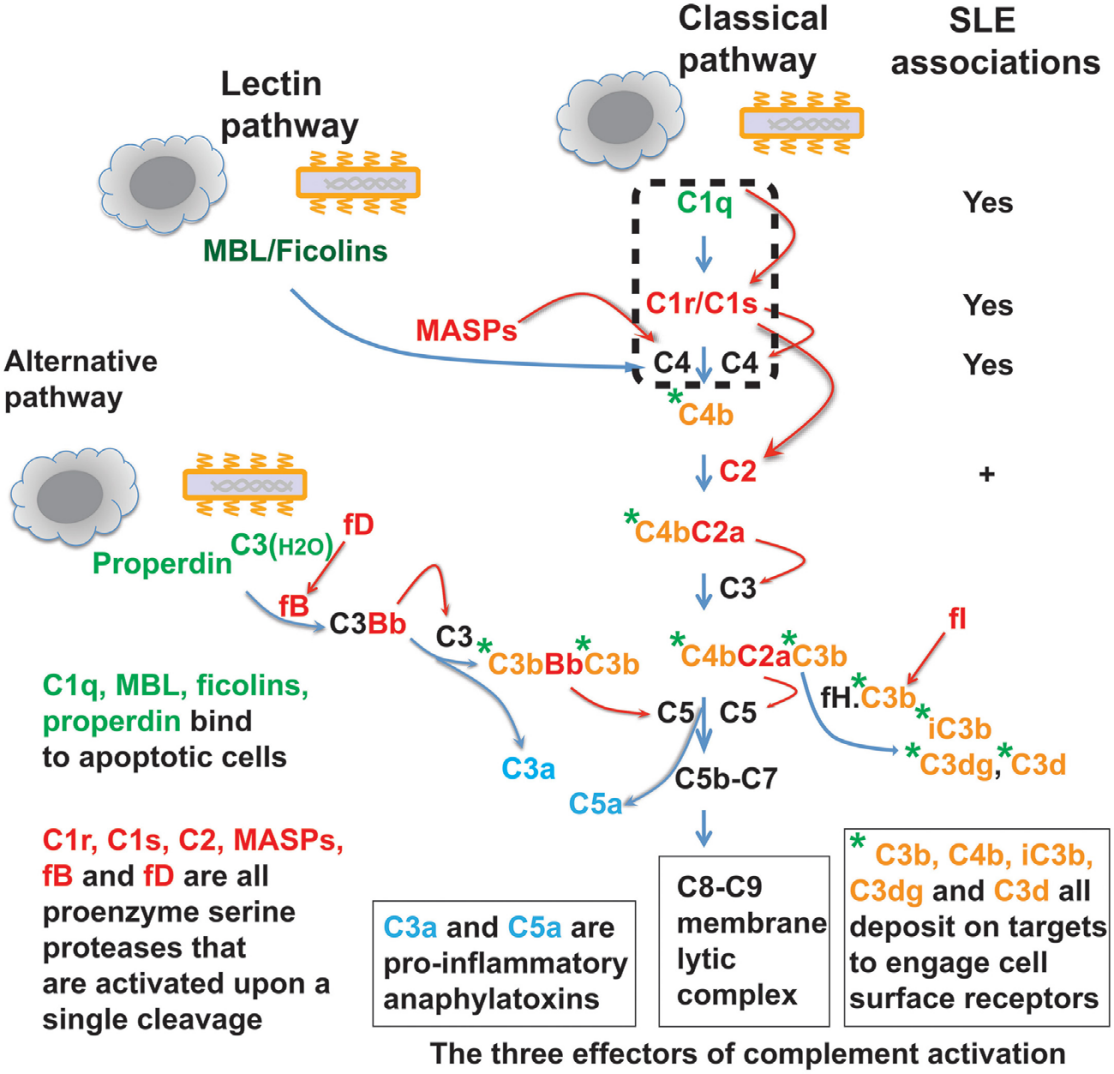
**The complement protein and protease network**. The complement system operates *via* three target recognition pathways: the classical, alternative, and lectin pathways. All pathways recognize microorganisms and apoptotic cells and the recognition subcomponents are in green. Upon its triggering *via* any of the three pathways, the complement acts through three effector reactions: the C8-C9 lytic or membrane attack complex, the soluble C3a and C5a anaphylatoxins (blue color), and surface-bound C3b, C4b, and further proteolytic fragment opsonins (orange color with green asterisk). All three pathways converge at the C3 component and complement reactions are essentially amplified through cascades of serine proteases (red color). MBL, mannose-binding lectin; MASPs, MBL-associated serine proteases; fB, factor B; fD, factor D; fI, factor I; fH, factor H. Homozygous deficiency of C1q, C1r/C1s, or C4 is causally associated with systemic lupus erythematosus (SLE) pathogenesis. Genetic C2 deficiency also increases risk for SLE and some other autoimmune diseases.

In invertebrates, complement takes primitive forms represented only by a few ancestral proteins and lacks the specificity and sophisticated regulatory mechanisms of the modern vertebrate complement system (1–4). In mammals and other higher vertebrates, the complement system is a complex protein network consisting of nearly 30 plasma proteins. Depending on the target ligands, the complement system can be activated *via* the classical, alternative, or lectin pathway (5, 6). In the case of microbial pathogens, each complement pathway is triggered through a specific mechanism of ligand recognition, and collectively, the three pathways empower this humoral system to defend against a broad range of microorganisms. Like the blood coagulation system, the complement system is orchestrated around serine proteases, which are sequentially activated and then cleave specific downstream complement proteins so as to amplify a cascade of reactions (2, 7, 8). These reactions generate proteolytic or lytic complexes, opsonins, and peptide anaphylatoxins leading to lysis, inflammation, and clearance of opsonized microorganisms (Figure 1) (5, 6). The complement serine proteases exhibit conserved active sites (2). However, these proteases are highly specific for substrate within the complement network, and this appears vital for the directional amplification of each pathway.

The complement system is commonly intended for host defense against microbial infections. Recent data suggest that various non-microbial exogenous and endogenous structures, such as apoptotic cells, may also trigger the complement pathways (Figure 1) (9–15). The effects of complement activation may also be delivered through a segment of the system rather than in its entirety. For example, the C1s protease apparently cleaves non-complement proteins including MHC class I molecule, insulin-like growth factor binding protein 5 (IGFBP5), Wnt receptor, and nuclear autoantigens (16–21). This suggests that, besides its well-defined roles in host defense, the C1 complex functions broadly, e.g., in tissue homeostasis and immune tolerance. In fact, invertebrates also utilize their limited repertoire of complement components to clear damaged cells as well as invading microorganisms (22, 23).

## The Classical Pathway is a Modern Pathway

During evolution from invertebrates leading up to higher vertebrates, animals experienced major genomic expansion through gene duplication and recombination, with higher vertebrates acquiring increased complexity in genomic composition, body plans, and physiological processes (24). The expansion of the complement system in higher vertebrates includes at least two aspects: the generation of paralogous complement elements and the formation of a new classical pathway. In invertebrates, ancestral complement elements were only found that were equivalent to the alternative and lectin pathways, including ancestral C3, factor B, mannose-binding lectin (MBL), ficolins, and MBL-associated serine proteases (MASPs) (3, 22). The modern C1 complex, i.e., the C1qC1r_2_C1s_2_ pentamer that defines the recognition component of the classical pathway, only appeared from jawed vertebrates when adaptive immunity also emerged.

**Figure 2:**
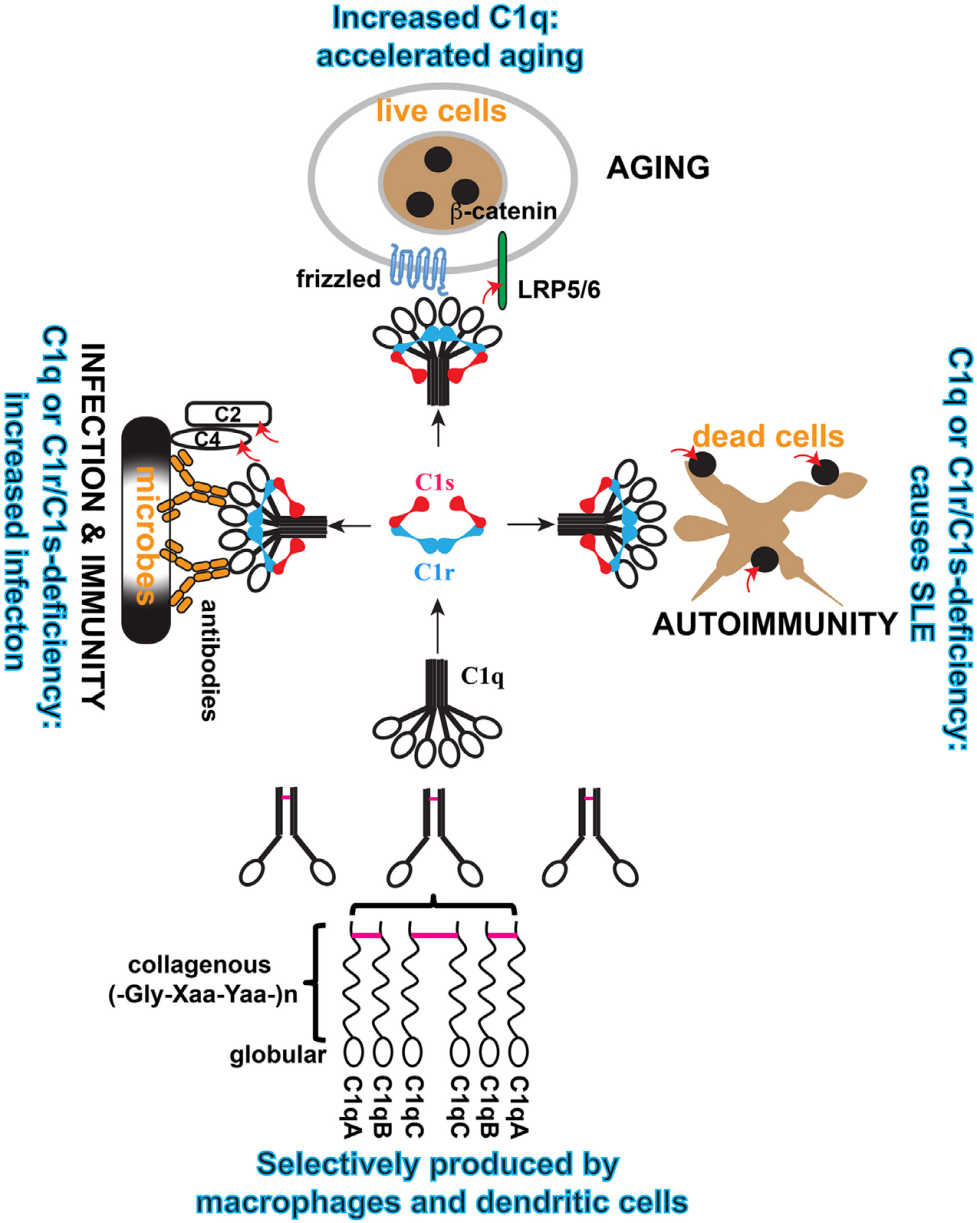
**Schematic illustration of the cellular origin of C1q, its assembly, and three distinct modular functions of C1 complex**. C1q is an abundant plasma protein. It can be produced broadly in tissues by macrophages and DCs, which also produce C1r/C1s. C1q is formed from three similar but distinct subunits, i.e., A, B, and C, and the order of assembly is illustrated. Inter-subunit disulfide bonds (purple bars) and the collagen-like helices defines the sorting of the three subunits within the C1q polypeptide. In the serum, C1q is associated with the serine protease proenzyme tetramer C1r_2_C1s_2_ to form the C1 complex. In this complex, C1q binds to diverse targets that activate the serine proteases and the proteases trigger effector reactions by cleaving specific substrate. Three physiological contexts are highlighted in which the C1 complex is known to play a role. When C1q binds to antibodies on microbial pathogens, the activated C1s cleaves complement C4 and C2 to initiate the proteolytic cascade. When C1q binds to Frizzled, activated C1s cleaves lipoprotein receptor-related protein 6 to cause canonical Wnt signaling and accelerated aging. When C1q binds to apoptotic cells, the activated C1s cleaves apoptotic cellular antigens to reduce autoimmunogenicity. The red arrows indicate C1s cleavage of the specific substrate.

Complement gene duplication and recombination are evident in higher vertebrates, e.g., factor B/C2, C3/C4/C5, and C6/C7/C8/C9 (3). Evidence that the C1r and C1s genes are relatively modern duplications is also suggested by their close genomic proximity and structural similarity (8, 25). This is even more striking with the three C1q subunit genes, i.e., C1qA, C1qB, and C1qC, which are clustered within a 30-kb genomic region separated by short intergenic sequences (26, 27) (Figure 2). The closest C1q-related protein in invertebrates is encoded by a single gene and the C1q-like protein recognizes carbohydrates rather than immunoglobulins (4). The emergence of the C1 complex or the classical pathway in higher vertebrates, which coincided with the appearance of the adaptive immune system, makes it a “modern” arm of the complement system that responds to antibodies and other self, non-self, and altered self targets.

## C1q Deficiency is a Strong Cause of Systemic Lupus Erythematosus (SLE) Pathogenesis

Genetic deficiency has been identified for many complement proteins and, in most cases, this increases susceptibility to infections (28, 29). Deficiency in some complement proteins is also associated with other pathological conditions and particularly strong associations were found between deficiencies in the early components of the complement classical pathway and the autoimmune disease SLE (29–34). The association is especially strong with homozygous C1 and C4 deficiencies. Functionally, C1q binding to ligands causes C1r and then C1s activation and the activated C1s cleaves C4 and then C2 to initiate the further downstream complement cascade (5, 6). C2 deficiency is more prevalent than C1 and C4 deficiencies, but it has substantially less effect and is also associated with other autoimmune diseases (31, 32). However, C1q, C1r/C1s, and C4 deficiencies cause predominantly SLE-like conditions.

In C1 and C4 deficiencies, the disease manifestations also deviate from that found in the larger SLE patient population. Typically, this specific group of SLE patients exhibit early disease onset and equal disease risks from both genders (30, 31, 35). SLE is otherwise a chronic disease that affects predominantly females at childbearing ages (36). How deficiency in each of these intimately related complement proteins, which define the classical pathway (Figure 1), causes SLE remains incompletely understood.

## Systemic Lupus Erythematosus

Clinical documentation of SLE disease has existed for more than a century. In 1948, Hargraves pioneered the mechanistic investigation of this disease by reporting the L.E. cell phenomenon, i.e., SLE patient serum caused polymorphnuclear leukocytes to bind or clump around autologous amorphous nuclear materials (37). The serum activity was later attributed to the γ-globulin fraction of the patient serum, presently known as autoantibodies reactive with chromatin or DNA (38–40). A pathogenic role for these autoantibodies became apparent when Tan et al. reported the asymptomatic appearance of anti-DNA autoantibodies, which disappeared during the ensuing disease flare when serum DNA antigen surged to complex with these autoantibodies (41). These autoantibodies are hallmarks in SLE pathogenesis and deposit in tissues leading to inflammatory tissue injury (42–44).

For a large majority of SLE patients, there is no definitive genetic explanation for the disease despite more than 50 SLE risk genes that have been identified (45). Most of these susceptibility genes are not specific for SLE and individually each risk gene has low-to-moderate effect on the disease (32). Known exceptions are genetic deficiencies of the intracellular exonuclease Trex1, and complement C1 and C4 (30–32). How deficiency in each of these complement proteins overrides the complex mechanisms governing host immunity and tolerance to cause this complex autoimmune disease is not fully understood. As anti-nuclear autoantibodies are pathogenic in SLE, understanding how these deficiencies cause anti-nuclear autoimmunity can provide greater insights into the underlying pathogenic mechanisms.

## Plasma C1q Accumulation is Associated with Accelerated Aging

While C1q deficiency causes autoimmunity, its elevated plasma levels signify accelerated aging. Aging is marked by a decline in tissue regeneration and repair, and in the number and dynamics of tissue stem or progenitor cells (46). At the molecular level, one observation is that progenitor cells exhibit elevated Wnt signaling in the aging tissue environment (47, 48). In aged mice, muscle stem cells exhibit increased tendency to fibroblastic differentiation (48). This was found to be conferred by a serum factor(s) in aged mice binding to the Frizzled family of cell surface receptors and causing Wnt receptor signaling (48). This Frizzled-binding protein was identified as C1q (48). Its serum level increased threefold (from 90 to 280 µg/ml) in old mice (20 months) as compared with young mice (2 months) (19).

**Figure 3:**
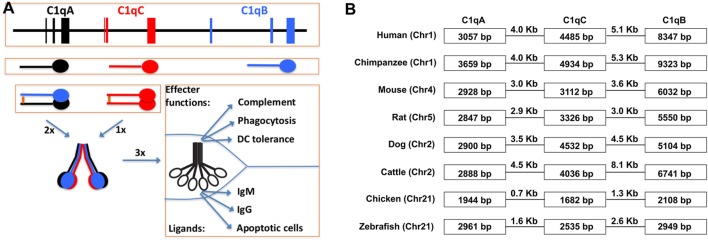
**Schematic illustration of C1q gene organization, gene transcription, and multimeric C1q assembly**. **(A)** The three human C1q genes span approximately 25 kb on human chromosome 1. The intergenic regions are 4.0 and 5.1 kb, respectively, which are not distinguishable in size from regular introns in the C1q genes. Each of the three C1q genes contains three exons and the transcribed peptides form disulfide-linked A-B hererodimers and C-C homodimers. Each C chain in the homodimer forms a collagen triple-helix with an A-B heterodimer, and hence, two triple-helices linked by the disulfide bond in the C-C dimer. Three such ABC-CBA twin helices associate non-covalently over the N-terminal ends to form the 18-polypeptide C1q molecule. The gC1q domains are often the ligand-binding sites for C1q and the collagen triple-helices associate with the C1r_2_C1s_2_ serine protease tetramer. **(B)** Conservation of the C1q gene organization in eight different animal species. The three C1q genes in chimpanzee occupied the largest genomic space which is approx. 27 kb. In chickens, the three C1q genes occupied merely 7.7 kb with intergenic sequences of 0.7 and 1.3 kb, respectively.

Mechanistically, C1q binding to the Frizzled receptors causes C1s activation and activated C1s cleaves the Wnt receptor protein low-density lipoprotein receptor-related protein 6 (LRP6) to trigger canonical Wnt receptor signaling (19) (Figure 3). The involvement of C4 and further downstream complement components are not defined. Nonetheless, this emphasizes the rather less-studied aspect of C1-mediated cleavage of proteins outside the complement network. C1s similarly cleaves MHC class I molecule, although the C1q ligands are not defined in this context (16, 17). It appears that activation of the complement classical pathway, which involves C1s cleavage of C4 and C2, is merely one of a number of effector mechanisms downstream of the C1 complex (Figure 3).

Besides a distinct decline in tissue regeneration and repair, aging is also characterized by systemic elevation of the inflammatory status (49, 50). In the elderly population, plasma pro-inflammatory cytokines, IL-6 and TNF-α, and the acute phase C-reactive protein (CRP) are chronically elevated. When young (<40 years) and aged (60–81 years) populations were compared in a series of age-related parameters, including muscle mass, plasma C1q, as well as plasma IL-6, TNF-α, and CRP, the young population had clearly lower plasma C1q (80.5 µg/ml) than the aged population (161 µg/ml) (51). Interestingly, after 12 weeks of supervised resistance training intervention, plasma C1q in the elderly group decreased substantially (89.3 µg/ml) with muscle mass being significantly increased, revealing an inverse correlation between plasma C1q level and muscle mass (51). The cause for plasma C1q accumulation in the elderly group and its reduction after training is unclear in this study and a causal relationship between plasma C1q and muscle mass was also not established (51). The overall conclusion was, however, in line with C1q contribution to accelerated aging as reported in mice (19).

## Mechanism of C1 Functional Diversity

The mechanisms for C1 complex function in the context of complement activation and Wnt receptor signaling have been clearly documented. However, mechanistic understanding of its involvement in SLE pathogenesis remains fragmentary (Figure 3). Genetic deficiencies in complement proteins generally increase susceptibility to infections but mostly lack the type of strong association with SLE pathogenesis that is observed with deficiencies of C1 and its immediate substrate C4 (28). This raises the possibility that SLE pathogenesis may be related to a modular C1 activity. Depending on what C1q recognizes, C1 may have effects through the C1r/C1s proteases on various molecular/cellular processes besides the complement system. C1 activation of Wnt receptor signaling is a good example of such a modular activity (19). The degradation of apoptotic cell debris is apparently another process involving a modular C1 complex function (Figure 3) (9).

Since the discovery of C1q binding to apoptotic cells (9), a significant body of work has been published revolving mostly around C1q opsonization of apoptotic cells and its regulation of immune tolerance. First, C1q binding to apoptotic cells opsonizes the cell debris for effective phagocytosis (10). Second, C1q binding contributes to the immunosuppressive nature of apoptotic cells (52, 53). Third, C1q modulates dendritic cell (DC) development to induce more prominent tolerogenic features in these antigen-presenting cells (54, 55). Last, C1q inhibits IFN-α production by DCs induced by SLE autoantibodies in the form of immune complexes (56–58). IFN-α is a SLE-pathogenic cytokine, which causes autoimmunity in patients following its therapeutic administration (59, 60). IFN-α is elevated in those SLE patients who register a chronically elevated signature of IFNα-stimulated gene transcription (61–63). Inhibition of IFN-α induction by C1q potentially contributes to protection against SLE pathogenesis.

Studies that evaluate the role of C1 proteases in these processes are lacking. In fact, how C1r/C1s deficiency also causes SLE has not been investigated. There are two hypotheses that are relevant to explaining how C1 and C4 deficiencies may cause autoimmunity (64, 65). A clearance hypothesis emphasizes on the induction of autoantibodies and autoimmunity by apoptotic cellular debris, which may accumulate due to impaired clearance or excessive cell death (64). A tolerance hypothesis emphasizes on the contribution of complement to promoting self-antigen delivery to primary lymphoid organs for an effective negative selection (65). Considering that C1s cleaves intracellular antigens, it can be highly significant that the C1 complex both opsonizes apoptotic cells through C1q for effective clearance and degrades apoptotic cellular antigens through C1 proteases. Without relying on the rest of the complement system, both processes can reduce the autoantigenicity of apoptotic cell debris.

C1q was initially found to bind to apoptotic blebs, but the spectrum of C1q ligands in apoptotic cells and their contributions to C1q recognition need further delineation (9, 66). C1q appears to bind multiple regions of apoptotic cells (20). In early apoptotic cells, C1q binds to peripheral structures; however, in late apoptotic cells, it binds predominantly to the core nuclear bodies, i.e., the nucleoli (20). With purified nucleoli, C1q not only binds to these nuclear bodies but also causes C1s activation and cleavage of nucleolar proteins, e.g., nucleophosmin-1 (NPM1) and nucleolin (20). Nucleoli are highly immunogenic and contain many autoantigens (67).

**Figure 4:**
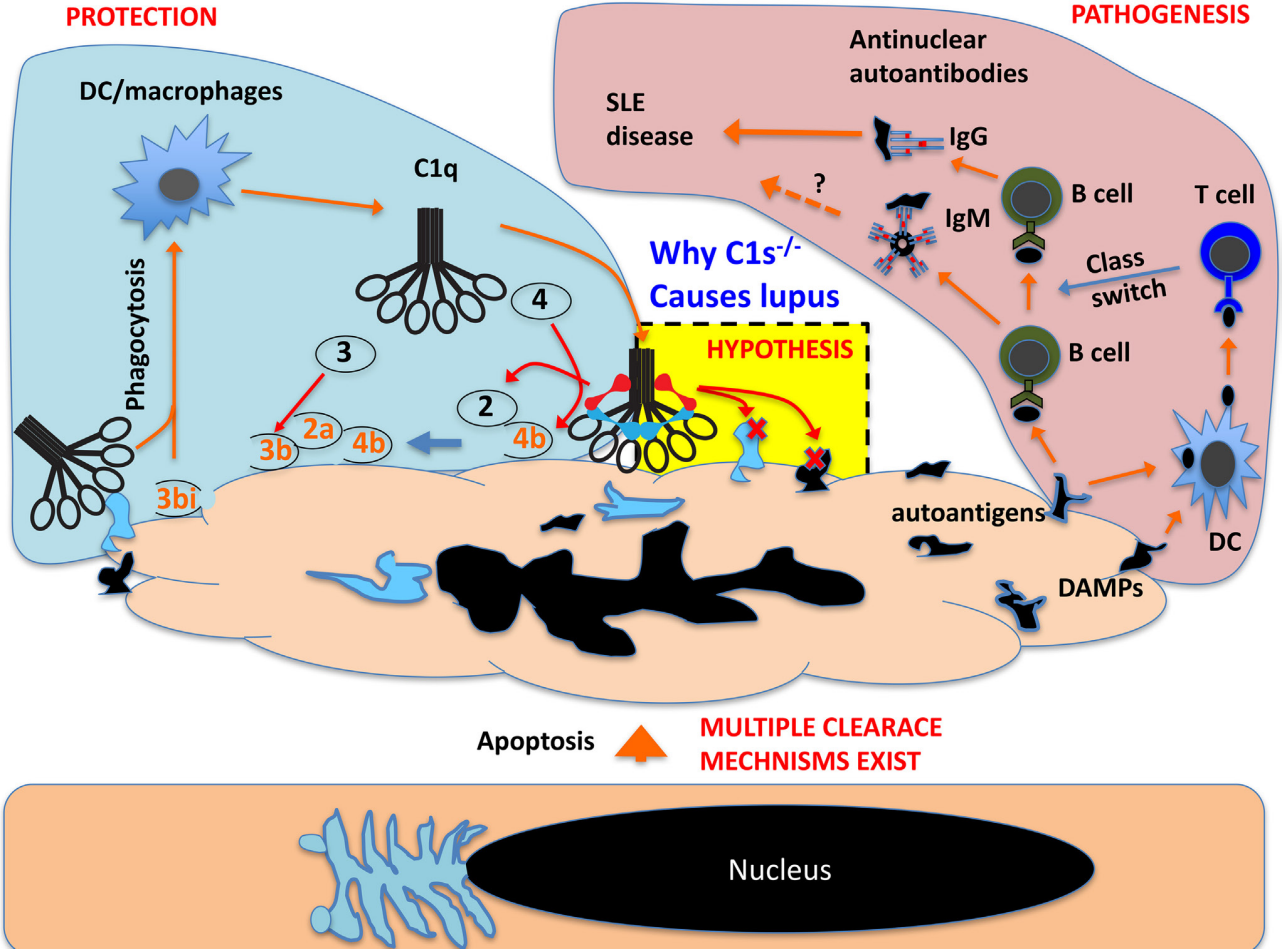
**Schematic proposal how C1 deficiency may cause systemic lupus erythematosus pathogenesis**. In live cells, the nucleus and other intracellular structures are compartmentalized and excluded from complement recognition. When cells undergo apoptosis, the nucleus and other cellular structures disintegrate and, in late apoptotic cells, these fragments are recognized by C1q, which opsonize apoptotic cells for phagocytosis. This can also cause C1r/C1s activation and the activated C1s could cleave its classic substrate C4 and C2 and produce complement opsonins for phagocytosis. C1s may also cleave numerous exposed nuclear and other cellular proteins that are otherwise autoimmunogenic (autoantigens) and cause B cell production of autoantibodies. C1s may also cleave cellular proteins that are otherwise pro-inflammatory danger-associated molecular patterns (DAMPs) and activate DCs to cause B cell production of pathogenic IgG autoantibodies. C1s may not inactivate all autoantigens but effective inactivation of DAMPs can abrogate class switch of autoantibodies from IgM to pathogenic IgG.

This reminds an important aspect in cell apoptosis, i.e., the intrinsic proteolytic/enzymatic dismantling of intracellular structures (68). During cell apoptosis, autoantigens are cleaved and partially inactivated by endogenous proteases (69). It is possible that during late stage apoptosis, exogenous proteases and other enzymes also contribute to the antigen dismantling process. C1q recognizes multiple intracellular regions during apoptosis, including the highly immunogenic nucleoli (20). In cooperation with endogenous proteases, C1 could contribute significantly to the effective protease trimming of dead cells required to prevent their immunogenicity (Figure 4) (70).

## C1r and C1s Substrateome

In the complement network, proteases are highly specific and this is essential to the directional propagation of the complement activation (5, 6). Outside the complement network, what other proteins may be cleaved by these proteases are rarely studied. With regard to C1s, it has been known for some time that it cleaves cell surface MHC class I and the secreted IGFBP5 (16–18). More recent addition to the list of non-complement C1s substrates includes LRP6, NPM1, and nucleolin (19, 20). In fact, the substrates of C1s can potentially be numerous based on bioinformatics predictions. Using a library of phage-displayed peptides that were designed based on the classic C1s cleavage sites on C4 and C2, Kerr et al. identified a list of C1s-cleavable peptide variants (21). Based on the conserved peptide framework, a formula was constructed that predicted numerous intracellular proteins as potential C1s substrate (21). NPM1 and nucleolin, which were found to be cleaved by C1 proteases, indeed contained multiple predicted C1s cleavage sites (20). The conjunction of a broad C1s substrateome with a diversity of C1q ligands makes the C1 complex a potentially multifaceted module that can function in a range of biological processes. C1s cleavage of intracellular proteins may be irrelevant to live cells, but this capacity could be important in the context of dead cell debris, reducing autoimmunogenicity by the inactivation of autoantigens and the destruction of danger-associated molecular patterns (DAMPs) (Figure 4). A recent example of this C1 protease function is the demonstrated C1s cleavage and inactivation of HMGB1, which is otherwise a nuclear DAMP (71).

## C1q

The functional versatility of C1q draws support from the modularity of its structures. C1q is a large, symmetrical, and delicate posttranslational assembly resulting from complex evolutionary innovations. At one stage, the complement system was defined by merely four identifiable components, C1–C4. In 1963, C1 was first separated into three distinct subcomponents, C1q and the two proteases C1r and C1s (72, 73). For C1q, biochemical analysis revealed three types of subunit polypeptides each containing a collagenous (Gly-Xaa-Yaa)n repeating sequence over the N-terminal half (74, 75). Similar collagen-like domains were later found in the N-terminal halves of collectins, ficolins, and some C1q/TNF-related proteins (CTRPs) such as adiponectin and saccular collagen (76–79). The collagenous regions of all these proteins form triple-helices and the C-terminal halves form globular (gC1q) domains that are clustered in three. The triple helices further conjoin at the extreme N-terminal regions to align 3–6 triple-helices in one final assembly (76, 77). In the overall “bundle of tulips” C1q assembly, the gC1q domains are peripherally extended as multivalent binding sites (74, 75). The six triple-helices in C1q form a scaffold for the tetrameric C1r_2_C1s_2_ protease complex (80). Binding of C1q to various ligands *via* the gC1q domain activates the C1r/C1s proteases and C1s triggers effector reactions through cleavage of specific substrate, which, in the complement classical pathway, are C4 and C2.

## The Structure of C1q Affords a Delicate Scaffold and Ligand-Binding Diversity

C1q is distinct from collectins, ficolins, and CTRPs in that it is assembled from more than one type of subunit polypeptide. The other proteins are considered largely homopolymers (76, 77). C1q is an 18-polypeptide macromolecule assembled equally from three similar but distinct subunit peptides, 6 × A, 6 × B, and 6 × C chains (74) (Figure 2). The C1q assembly is partially stabilized by disulfide bonds and, under denaturing conditions, the molecule crumbles into two basic structural identities, an A-B heterodimer and a C-C homodimer that are linked through N-terminal disulfide bonds (Figure 2). One C-C and two A-B dimers form two triple helices over the collagen-like regions (ABC-CBA) and C1q is assembled non-covalently from three such ABC-CBA structures (Figure 2). Therefore, despite the presence of three C1q genes, only one type of C1q is assembled. The collectins, ficolins, and CTRPs are, however, products of single genes (76, 77).

What prevented the formation of three different homopolymeric C1q molecules is not understood. The combination of divergent subunits, i.e., A, B, and C, and their extensive polymerization in C1q offers, besides a scaffold to embrace the C1r_2_C1s_2_ tetramer, diversity and multiplicity of binding sites for a broad ligand repertoire. The heterotrimeric congregation of the three globular head modules (ghA, ghB, and ghC) yielding gC1q domain at the C-termini is independent of the N-terminal triple-helix (81). The three different globular head modules in the cluster exhibit differential binding preferences toward known C1q ligands (82, 83).

## The Broad Tissue Origins of C1q and Its Ultimate Plasma Destiny

A dominant source for plasma complement proteins, including C1r and C1s, are hepatocytes in the liver, but C1q is one exception for its extrahepatic origins (84). C1q was initially found produced by macrophages (85). It was later found to be produced by tissue and cultured DCs as well (86, 87). Studies on C1q gene promoters revealed active *cis*-acting elements for transcription factors PU.1 and IRF8 (26). PU.1 and IRF8, especially PU.1, is a key transcription factor that defines the macrophage and DC lineage of hematopoietic development (88). Tenner and colleagues recently clarified that, in the brain, C1q is also produced by local tissue macrophages, the microglia (89). Therefore, C1q could have evolved first as an effector molecule in macrophages or ancestral phagocytes and its association with the C1r/C1s proteases in the form of C1 complex represents a secondary evolutionary innovation.

Macrophages and DCs populate many tissues and are poorly represented in the blood circulation (90, 91). Monocytes are blood precursors of some tissue macrophages, but these cells only start to produce C1q upon differentiation into macrophages (92). How the broad and heterogeneous tissue origin of C1q and its steady plasma levels are regulated is not fully understood. Tissue macrophages, which orchestrate inflammation and antigen presentation as well as scavenge tissue debris and microorganisms, are responsive to diverse stimuli (93, 94). The complement system is concentrated in the blood and is actively recruited to sites of tissue infections or injuries. The macrophage/DC origin of C1q appears to ensure its steady state tissue distribution. Macrophages also produce C1r/C1s proteases (84). DCs also broadly populate tissues, albeit at a lower density, and also produce C1q, C1r, and C1s (86, 87, 95). This mode of C1q and C1r/C1s production stresses an important C1q or C1 function in sterile tissue homeostasis and other molecular/cellular processes.

## Plasticity in C1q Production

Macrophages express a broad repertoire of scavenging and signaling receptors and exhibit a high degree of plasticity in differentiation and activation. This is reflected in the heterogeneity of tissue macrophages in their morphology and effector molecule production (91). As previously summarized, C1q production by macrophages also vary in response to microbial structures, cytokines, hormones, and drugs (66, 96). Overall, microbial structures tend to inhibit C1q production and corticosteroid hormones tend to enhance it (66). With respect to cytokines, IFN-α appears to inhibit C1q production (87), whereas IFN-γ increases C1q production by DCs/macrophages (26, 97). Local and temporal tissue fluctuation in C1q production may not prominently alter plasma C1q levels, but it can impact on local tissue homeostasis, immunity, and tolerance. This can also be of great importance in the microenvironment of tumor, where C1q seems to have a tumor-promoting function (98).

## Does C1r/C1s Cleave Other C1q-Targeted Proteins?

Besides IgG and IgM, many other protein ligands have been identified for C1q (66, 99). These C1q ligands, including soluble, cell surface, normal extracellular matrix, and pathogenic amyloid proteins, often activate C1r/C1s and the complement classical pathway. It has, however, not been addressed whether the activated C1r/C1s proteases also cleave these C1q ligands or proteins near these ligands as they cleave LRP6, IGFBP5, MHC I, NPM1, and nucleolin (16–20). In some pathophysiological contexts, C1q functions were interpreted without specific consideration to its ligands. In the postnatal central nervous system, C1q is localized to synapses and contributes to synapse elimination resembling the disposal of dead cells, which is important for the maturation of neuronal connectivity and functions (100, 101). How C1q causes the selective dismantling of synapses is unclear, but it is tempting to suggest C1q binding to selective neuronal contexts and possible involvement of C1r/C1s-mediated molecular cleavage or cell signaling as observed with the Wnt receptor (19). In excess, the same C1q-mediated synapse elimination could accelerate neurodegeneration related to aging and neuropathology (102, 103).

The scrapie pathogen, prion protein, is another C1q ligand (104, 105). C1q deficiency reduces scrapie pathogenesis (106, 107). To what extent the complement classical pathway may be involved in prion-mediated pathology is incompletely defined, but C4 is apparently activated on prion proteins and C3 depletion also reduced scrapie pathogenesis (104, 107). As C1q, C3, and C4 are all potent opsonins, a prevalent explanation is their involvement in prion transmission from the gut to the central nervous tissues. The role of activated C1r/C1s proteases in scrapie pathogenesis has not been considered.

C1q is also produced in the placenta (108, 109). At this feto–maternal interface, it was shown to mediate trophoblast invasion of the maternal decidua (108). Mechanistically, C1q was found to interact with decidual stroma, to activate trophoblast signaling, and to mediate trophoblast adhesion and migration (108). Whether C1r/C1s might play a role in this context is again unclear.

## Concluding Remarks

The complement system is an intimate proteolytic cascade responding to diverse triggering factors. In infections or injuries, the full impact of its activation is realized by three closely related effector reactions: inflammation, opsonization, and lysis (5, 6). The C3a and C5a anaphylatoxins recruit and activate phagocytes and other inflammatory leukocytes at sites of tissue infections or injuries. The membrane attack complexes cause cellular lysis. The C4b, C3b, and the further proteolytic fragments opsonize complement-reacted targets for effective phagocytic clearance (Figure 1). However, this article highlights that C1 complex may function as a module, independent of the rest of the complement network, to participate in other molecular/cellular processes.

Serine proteases are core components of the complement infrastructure and their sequential activation is at the heart of the formation of hierarchical proteolytic or lytic protein complexes. In the context of the complement network, these are highly specific proteases, e.g., C1r only cleaves C1s and C1s only cleaves C4, C2, and C1 inhibitor. The finding that the C1 proteases also cleave a growing list of non-complement proteins, including LRP6, MHC I, IGFBP5, NPM1, and nucleolin, supports a multifaceted, modular function for C1 complex. In this functional C1 module, C1q recognizes targets in various molecular/cellular processes and the C1r/C1s proteases bring about the effects by cleaving substrate in these molecular/cellular processes. Modular functions may also be found in other complement proteases such as factor B and MASPs.

## Author Contributions

JL initiated the article and contributed to the framework and major details of the final version. UK provided critical and substantial inputs to the overall scope and detailed content. This is a joint project by the two authors.

## Conflict of Interest Statement

The authors declare that the research was conducted in the absence of any commercial or financial relationships that could be construed as a potential conflict of interest.

## Abbreviations

IGFBP5, insulin-like growth factor-binding protein 5; MBL, mannose-binding lectin; MASP, MBL-associated serine protease; SLE, systemic lupus erythematosus; LRP6, low-density lipoprotein receptor-related protein 6; CRP, C-reactive protein; DC, dendritic cell; NPM1, nucleophosmin-1; CTRP, C1q/TNF-related protein.
